# Inhibition of VEGF expression and corneal neovascularization by shRNA targeting HIF-1α in a mouse model of closed eye contact lens wear

**Published:** 2012-04-06

**Authors:** Peng Chen, Hongmei Yin, Ye Wang, Yao Wang, Lixin Xie

**Affiliations:** 1 Qingdao University Medical College, Qingdao, China; 2 State Key Laboratory Cultivation Base, Shandong Provincial Key Laboratory of Ophthalmology, Shandong Eye Institute, Shandong Academy of medical Sciences, Qingdao, China

## Abstract

**Purpose:**

Inappropriate contact lens (CL) use and care often lead to corneal neovascularization (corneal NV). We used mouse eyes which wore CL as the animal model to assess the reason for corneal NV with CL wear. The similar and overlapping activity of vascular endothelial growth factor (VEGF) and the potent angiogenic hypoxia-inducible factor 1α (HIF-1α) called for a study of the temporal relationship in the expression of these two autocoids. We determined the time dependent expression of HIF-1α and correlated it to that of VEGF expression in the mouse model of closed eye with CL wear.

**Methods:**

Mouse eyes were fitted with CL followed by a silk suture tarsorrhaphy. The anterior surface was analyzed at 4, 7, and 10 days after tarsorrhaphy by slit lamp and corneal NV. HIF-1α and VEGF levels were measured by reverse transcription PCR, western blotting and immunofluorescence with specific primers and antibodies. We used shRNA targeting *HIF-1α* to substantiate the link between HIF-1α, VEGF expression, and angiogenesis in the CL wear model.

**Results:**

Corneal NV scores increased in a time dependent manner in the model of closed eye CL induced hypoxic injury. Corneal epithelial HIF-1α and VEGF expression increased in a time dependent manner. The prolonged hypoxic state brought by closed eye CL wear induced a time dependent neovascular response which was significantly attenuated by *HIF-1α* specific shRNA but not by nonspecific shRNA. Both HIF-1α and VEGF levels were reduced significantly in corneal homogenates from eyes treated with the *HIF-1α* specific shRNA.

**Conclusions:**

The present study documented the increased expression of HIF-1α in the corneal epithelium during CL wear. It also demonstrated the presence of VEGF in the corneal epithelium and its increased expression in this model. Altogether, the results of this study raised the possibility of interaction between HIF-1α and VEGF, in mediating the neovascularization response induced by the prolonged hypoxic state brought about by closed eye CL wear. The results strongly implicated corneal HIF-1α as a component of the inflammatory and neovascular cascade initiated by hypoxic and further suggested that HIF-1α was a proximal regulator of VEGF expression in this model.

## Introduction

Since contact lens (CL) was introduced in the USA in the 1950s, advances in CL technology have enabled great improvements to be made in CL safety and comfort. As the lens materials have improved, the popularity of CL wear has increased; currently, a large number of people, from children to the elderly, wear CL. With an increased population of CL wearers, complications related to inappropriate CL use and care are of serious concern. Among these complications, corneal neovascularization (corneal NV) has frequently been reported [1].

In general, the normal cornea is a nonvascular tissue whose metabolism for maintaining transparency is dependent on oxygen and nutrients. When a CL is worn, there are two major oxygen delivery systems regulating the oxygen tension at the tear-lens interface: the diffusion of oxygen from the air through the lens material and the pumping of oxygenated tears beneath the lens during lens movement by blinking [2]. Prolonged hydrophilic CL wear induces a state of hypoxia to the corneal surface frequently associated with inflammation and neovascularization of the corneal surface. Hypoxia is believed to be the principle contributor to adverse effects of CL wear [3,4].

A master regulator of the hypoxic response is the transcription factor hypoxia-inducible factor 1 (HIF-1), which consists of an α-subunit whose proteasomal degradation and thus relative abundance are regulated by oxygen tension, and a constitutively expressed β-subunit [5]. HIF-1 transactivates the expression of proangiogenic genes in response to hypoxic conditions and plays important roles in vasculogenesis and angiogenesis [6,7]. Binding of HIF-1 to the hypoxia response element of the vascular endothelial growth factor (*VEGF*) promoter results in transcriptional activity [8]. VEGF, a potent and specific mitogen for vascular endothelial cells, is a critical mediator of corneal NV. Animal studies have shown that *VEGF* overexpression is sufficient to induce corneal NV in the eye [9,10], whereas inhibition reduces this effect [11]. *VEGF* is also expressed in laser-induced corneal NV [12] and surgically excised corneal NV membranes [13], and multiple preclinical and clinical trials have proved that anti-VEGF strategies were effective as potential therapeutic agents for the treatment of corneal NV [14].

Because HIF-1α activates the transcription of *VEGF*, which is required for corneal NV, it is possible that hypoxia may mediate corneal NV through the induction of *HIF-1α* and *VEGF*. However, little is known about this; even less is known about the upstream signaling events that are activated by hypoxia and that mediate its effects in corneal NV. The similar and overlapping activities of VEGF and HIF-1α calls for a study of the temporal relationship in the expression of these two autocoids. The current study was undertaken to further substantiate the cause and effect relationship between *HIF-1α*, *VEGF* expression, and neovascularization in a mouse model of closed eye with CL wear. We determined the time dependent expression of *HIF-1α* and correlated it to that of *VEGF* expression in the model. Herein, we demonstrated that shRNA targeting corneal *HIF-1α* inhibited corneal NV further supporting the role of *HIF-1α* as an angiogenic pathway in the cornea.

## Methods

### Design of gene targets and shRNA

ShRNA sequences were derived from the coding sequence of mouse *HIF-1α*. The RNAi-Ready pSIREN-RetroQ vector (Clontech, Palo Alto, CA) was used. Double stranded (ds) oligonucleotides encoding *HIF-1α* shRNA were chemically synthesized and contained 5′-BamHI and 3′-EcoRI sites for insertion. The *HIF-1α* shRNA sequences were as follows: RNAi-A, 5′-gat ccG ATG CTT ACA CAC AGA AAT GTA GTG CTC CTG GTT GCA TTT CTG TGT GTA AGC ATC ttt ttt g-3′; RNAi-B, 5′-gat ccG GTC ACC ACA GGA CAG TAC ATA GTG CTC CTG GTT GTG TAC TGT CCT GTG GTG ACC ttt ttt g-3′. The sequence used as control did not encode for any meaningful fragment 5′-gat ccG CAA AGG TCG ACT ACC ACG ATA GTG CTC CTG GTT GTC GTG GTA GTC GAC CTT TGC ttt ttt g-3′). Oligonucleotides were inserted into the vector using the BamHI and EcoRI sites immediately downstream of the U6 promoter. The recombinant vectors were named as HIF-1α RNAi-A, HIF-1α RNAi-B, and Vehicle-RNAi.

### shRNA transfection and hypoxia induction in vitro

Simian virus 40-immortalized human corneal epithelial cells (HCECs) were provided by Choun-Ki Joo (The Catholic University of Korea, School of Medicine, Seoul, Korea). The cells were cultured in DMEM/F-12 (1:1) media, 5% fetal bovine serum (FBS; Gibco-BRL, Grand Island, NY), 5 μg/ml insulin (Sigma, St. Louis, MO), 0.1 ng/ml cholera toxin (EMD Biosciences, San Diego, CA), 10 ng/ml human epidermal growth factor (hEGF; R&D Systems, Minneapolis, MN), and 0.5% dimethyl sulfoxide (DMSO; Sigma) in a humidified 5% CO_2_ incubator at 37 °C [15]. HCECs were grown to 80% confluence. The recombinant vectors were transfected into HCECs using Effectence Transfection Reagent (Qiagen GmbH, Hilden, Germany) according to the manufacturer's protocol. Thirty-six hours after transfection, hypoxia was induced in the cells by the addition of CoCl_2_ to a final concentration of 1 mM in each well. Five hours after CoCl_2_-induction the supernatant was removed from all wells and adherent cells were collected. For detecting expression of HIF-1α, western blotting was performed on the collected cells. Experiments were repeated three times.

### Animals and samples

All animal experiments were performed in accordance with the guidelines of the Association for Research in Vision and Ophthalmology Statement for the Use of Animals in Ophthalmic and Vision Research. BALB/C mice of both sexes (male:female=1:1), 8–10-weeks old, were purchased from the Institution of Laboratory Animal Sciences (Chinese Academy of Medical Sciences, Beijing, China) and were housed at the Shandong Eye Institute Animal Facility. Briefly, the mice were anesthetized intraperitoneally with ketamine (37.5 mg/ml) and xylazine (1.9 mg/ml). Proparacaine hydrochloride (0.5%) was used for topical anesthesia. Only the right eye (OD) of each mouse was used for CL wear; the left eye (OS) was undisturbed.

The Etafilcon A lens (Acuvue, Jacksonville, FL) were prepared with a microkeratome and punched with a 3.5 mm trephine. Following the creation of some standard 3.5 mm lenses, the small hydrophilic CL was placed onto the proptosed OD in a stacked fashion (2 lenses, one on top of the other) and the eye was gently reposited. To keep the lenses in place, 5–7 interrupted 10–0 silk sutures were placed through the superficial tarsus of both eyelids with care taken not to penetrate the conjunctival tissues. Immediately after surgery, 0.3% ofloxacin eye ointment (Tarivid; Santen, Osaka, Japan) was administered to the palpebral margin.

For gene therapy, preparations were injected into the subconjunctival site 1 h before surgery of the CL wear. Briefly, a needle pierced into the conjunctiva from the corneoscleral limbus, and 5 μl of shRNA (1 μg/μl) or saline was injected. Control animals were only treated with the surgery of the CL wear. Each group contained ten BALB/C mice. At 4, 7 and 10 days after surgery mice were anesthetized and the eyelid sutures and lenses were removed. Cornea photographs were taken with a camera mounted on the slit-lamp microscope (Zeiss, Jena, Germany) under the same magnification. Neovascularization of each cornea, as described by others [16,17], was determined by a blind examiner to minimize the observer’s bias. Briefly, the neovascularization score=(distance from the limbus to the end point of the cornea neovascularization/distance from the limbus to the center)/0.17. Each cornea received an exact score. Scores were concluded representatively as follows: 0 (no visible vessels in the cornea), +1.5 (1/4 distance to center), +2 (1/3 distance to center), +3 (1/2 distance to center), +4 (2/3 distance to center), +4.5 (3/4 distance to center), and +6 (vessels reach center).

### Reverse-transcription PCR

Total RNA was prepared from each cornea, using the NucleospinRNA kits (BD Biosciences, Palo Alto, CA), and reverse transcribed into first-strand cDNA, using Primescript™ First-Strand cDNA Synthesis kit (TaKaRa, Dalian, China). Each group contained three corneal tissues. Gene-specific cDNA fragments were amplified with DNA polymerase (Tiangen, Beijing, China). The expression of genes was normalized to glyceraldehyde-3-phosphate dehydrogenase (*GAPDH*). The primer sequences for these reactions were shown in Table 1. PCR amplification products were analyzed by agarose gel electrophoresis.

**Table 1:** The sequences and size product for RT–PCR primers.

**Gene name**	**Primer sequences (F)**	**Primer sequences (R)**	**Product length**
*GAPDH*	GGTGAAGGTCGGTGTGAACGGA	TGTTAATGGGGTCTCGCTCCTG	246 bp
*HIF-1α*	GGTATTATTCAGCACGACTT	GGAGACATTGCCAGGTTTAT	323 bp
*VEGF*	GAGCAGAAGTCCCATGAAGTG	CATGGTGATGTTGCTCTCTGA	213 bp
*IL-1β*	GCCCATCCTCTGTGACTCAT	AGGCCACAGGTATTTTGTCG	230 bp
*MMP-2*	CCCGATCTACACCTACACCAA	AAACCGGTCCTTGAAGAAGAAC	217 bp
*MMP-9*	CGTCGTGATCCCCACTTACTA	AAGATGAACGGGAACACACAG	237 bp

### Antibodies and western blotting

Total protein was prepared from each cornea, using radio immunoprecipitation assay (RIPA) buffer (50 mmol/l Tris pH 7.4, 150 mmol/l NaCl, 1% Triton X-100, 1% sodium deoxycholate, 0.1% sodium dodecyl sulfate [SDS], sodium orthovanadate, and sodium fluoride; Galen, Beijing, China), and quantified. Protein (50 μg in 15 μl loading buffer) was resolved in 10% sodium dodecyl sulfate PAGE (SDS–PAGE) gel and then transferred to a polyvinylidene difluoride membrane (Millipore, Billerica, MA). The blots were blocked in 5% nonfat dry milk dissolved in Tris-buffered saline Tween-20 (TBST; 20 mmol/l Tris, pH 7.5, 0.5 mmol/l NaCl, 0.05% Tween-20) for 1 h and then incubated with the primary antibody in TBST for 1 h, followed by incubation with horseradish peroxidase-conjugated secondary antibody (Amersham Biosciences, Uppsala, Sweden) for 1 h. All incubations were done at 25 °C, and three washes with 10 ml TBST were applied between each step. The membranes were then developed with SuperSignal West Femto Maximum Sensitivity substrate (Pierce Biotechnology, Rockford, IL) and exposed to X-ray film (Kodak, Rochester, NY). The immunoreactive bands were quantified using National Institutes of Health (NIH) Image 1.62 software (NIH, Bethesda, MD).

Primary antibodies included anti-VEGF antibody (Abcam, Cambridge, MA), anti-HIF-1α antibody (abcam, Cambridge, MA), anti-GAPDH antibody (Kangchen, Shanghai, China), anti-phosphorylated nuclear factor-kappa B (P-NF-κB) antibody, and anti-NF-κB antibody (Cell Signaling Technology, Danvers, MA). All the experiments reported in this study were performed three times, and the results were reproducible. For each sample, the levels of proteins of interest were normalized to that of GAPDH.

### Immunofluorescence and immunohistochemistry

Eyeballs were snap-frozen in optimal cutting temperature (OCT) compound (Sakura Finetechnical, Tokyo, Japan). Cryosections (6 μm) were prepared from OCT-embedded eyeballs and were fixed in ice-cold acetone for 10 min. The sections were blocked with 10% normal goat serum for 15 min and stained with anti-VEGF antibody and anti-HIF-1α antibody (abcam, Cambridge, MA) overnight at 4 °C. Sections were washed and stained for 30 min at 37 °C with rhodamine-conjugated goat anti-mouse immunoglobulin G (IgG) secondary antibody (Santa Cruz, Inc., CA). After counterstaining with 4,6-diamidino-2-phenylindole (DAPI), the stained sections were viewed under an Eclipse TE2000-U microscope (Nikon, Tokyo, Japan). Negative controls were performed by omitting primary antibodies.

Eyeballs were fixed in 10% buffered formalin, and embedded in paraffin. Paraffin sections of 4 μm in thickness were deparaffinized, rehydrated, and stained with anti-P-NF-κB antibody using routine protocols.

### Statistical analysis

Data are presented as mean±standard deviation (SD). The differences between control and experimental conditions were analyzed using the one-way ANOVA (ANOVA) and Student-Newman-Keul's (SNK) test. The statistical differences were evaluated by SPSS 11.5 software (SPSS Inc., Chicago, IL), and a p<0.05 was considered significant.

## Results

### CL wear induced corneal NV

**Figure 1:**
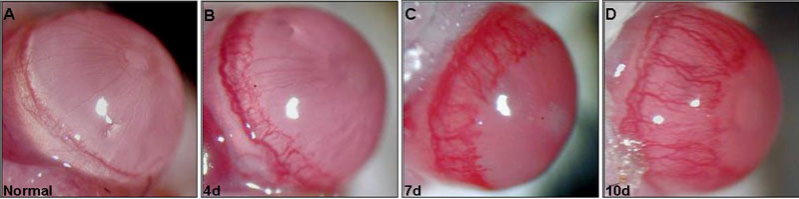
Representative pictures depicting corneal NV in eyes at days 4, 7, and 10 after CL wear.

Corneal NV was apparent in eyes with CL wear as early as 4 days after its placement (Figure 1B). The neovascular response increased with time encompassing larger area of the cornea by day 7–10 after CL wear (Figure 1C, D). Quantitative analysis showed that the neovascular response to CL wear in eyes receiving the CL wear was upregulated by 1.56 and 2.36 fold on day 7 and 10 over the neovascular response respectively by day 4 of CL wear (Table 2).

**Table 2:** Quantitative analysis of corneal NV 4, 7 and 10 days after CL wear (mean±SD).

** **	** **	Neovascularization score*			** **
**Groups**	**Number of eyes**	**Maximum**	**Minimum**	**Mean**	**p (versus normal)**
Normal	10	0	0	0	－
4d	10	2.14	0.98	1.53±0.35	0.000
7d	10	3.24	1.67	2.39±0.48	0.000
10d	10	5.49	1.81	3.59±1.10	0.000

Neovascularization was scored according to the distance from the limbus to the farthest end of the new vesicles: 0 (no visible vessels in cornea), +1.5 (1/4 distance to center), +2 (1/3 distance to center), +3 (1/2 distance to center), +4 (2/3 distance to center), +4.5 (3/4 distance to center) and +6 (vessels reach center).

**Figure 2:**
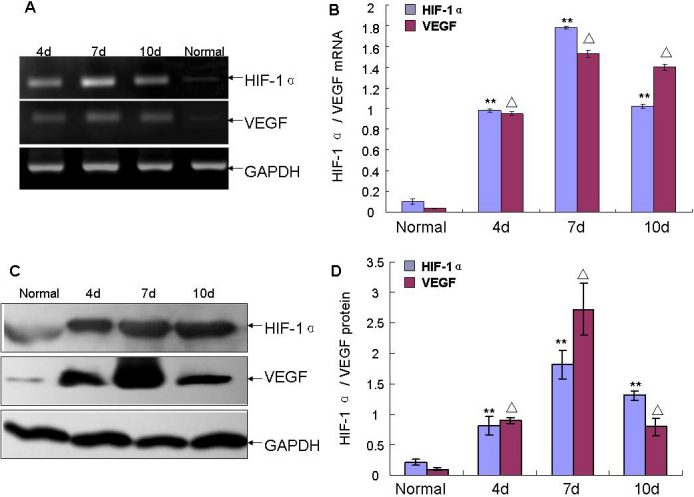
Relationship between the duration of closed eye CL wear, corneal HIF-1α and VEGF levels. **A**: Corneal RNA was extracted and RT–PCR was performed with *HIF-1α*, *VEGF*, and *GAPDH* specific primers. **C**: western blotting analysis was performed as described in Methods. Corneal HIF-1α and VEGF levels were normalized to that of GAPDH. **B**, **D**: Densitometry analysis expressed in arbitrary units as the mean±SD, n=3, **p<0.01, as compared with HIF-1α of normal cornea. △p<0.01, as compared with VEGF of normal cornea.

**Figure 3:**
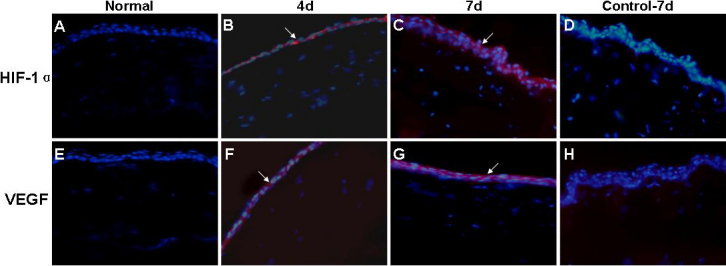
Immunofluorescence staining of HIF-1α and VEGF in cornea at day 4 and 7 after CL wear. The arrows indicated the positive staining of HIF-1α and VEGF in the corneal epithelium of each group. **A**: HIF-1α expression in normal cornea. **B**: HIF-1α expression in cornea at day 4. **C**: HIF-1α expression in cornea at day 7. **D**, **H**: Control cornea at day 7. **E**: VEGF expression in normal cornea. **F**: VEGF expression in cornea at day 4. **G**: VEGF expression in cornea at day 7. Magnification: 400×.

The changes of HIF-1α and VEGF abundance were confirmed by RT–PCR at mRNA level (Figure 2A,B) and by western blotting (Figure 2C,D) or immunofluorescence (Figure 3) at the protein level. Collectively, these results implied that HIF-1α and VEGF were involved in the pathogenesis of CL-induced corneal NV.

### In vitro efficacy of *HIF-1α* shRNA

**Figure 4:**
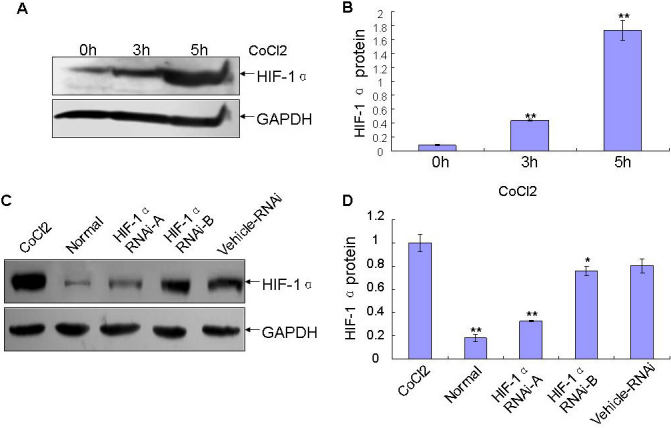
Inhibition of HIF-1α induced by CoCl_2_ by *HIF-1α* specific shRNA in HCECs. **A**: HIF-1α was upregulated after 3 h and 5 h induction of CoCl_2_ over normal corneas. **B**: Quantitative analysis of HIF-1α protein expression in HCECs. Results were mean±SD, n=3, **p<0.01 as compared with 0 h group. **C**: western blotting analysis of HIF-1α expression in HCECs treated with shRNA targeting *HIF-1α* (RNAi-A, RNAi-B) and nonspecific shRNA (Vehicle- RNAi) and incubated with CoCl_2_. **D**: Quantitative analysis of HIF-1α expression from panel **C**. Results are mean±SD, n=3, *p<0.05, **p<0.01 as compared with CoCl_2_ group.

HCECs exhibited very low levels of *HIF-1α* expression. *HIF-1α* was upregulated by 5.5 and 21.6 fold after 3 h or 5 h induction of CoCl_2_ over normal cornea (Figure 4A,B). Therefore, HCECs overexpressing *HIF-1α* induced by CoCl_2_ for 5 h were used to evaluate the efficacy of shRNA targeting *HIF-1α*. Western blotting analysis revealed that both *HIF-1α* RNAi-A and *HIF-1α* RNAi-B substantially attenuated protein levels of HIF-1α induced by CoCl_2_ (Figure 4C). *HIF-1α* RNAi-A attenuated endogenous HIF-1α protein levels with a maximum blockage of 60% compared with the Vehicle-RNAi (Figure 4C,D).

### *HIF-1α* shRNA inhibited corneal NV

**Figure 5:**
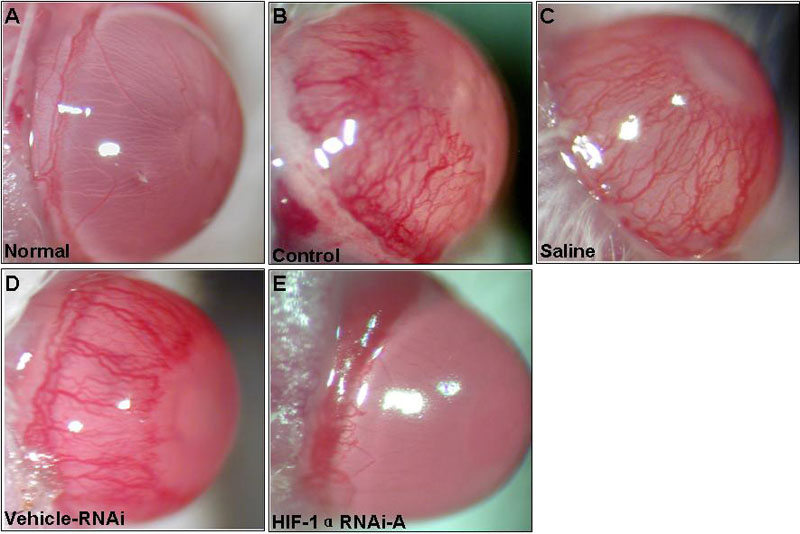
Effect of shRNA treatment on corneal NV. Representative pictures depicting corneal NV in normal and control eyes (**A**, **B**) and in eyes receiving subconjunctival injections of saline (**C**), nonspecific shRNA (Vehicle- RNAi; **D**) or *HIF-1α* specific (RNAi-A; **E**) at day 10 after CL wear.

Corneal NV was apparent in eyes with CL wear on day 10 after its placement. In eyes treated with saline, the neovascular response increased with larger area of the cornea by day 10 after CL wear (Figure 5C). Similar neovascular response was observed in eyes treated with Vehicle-RNAi (Figure 5D). In contrast, corneal NV was markedly reduced in eyes receiving subconjunctival injections of *HIF-1α* specific shRNA (RNAi-A) (Figure 5E) as compared to eyes treated with the vehicle-RNAi or saline. Quantitative analysis showed that the neovascular response to CL wear in eyes receiving the *HIF-1α* RNAi-A injection was inhibited by about 69% and 72% at day 10, respectively, when compared with Vehicle-RNAi or saline group (Table 3).

**Table 3:** *HIF-1α* shRNA on corneal NV after 10 days of CL wear (mean±SD).

** **	** **	Neovascularization score*			** **
**Groups**	**Number of eyes**	**Maximum**	**Minimum**	**Mean**	**p (versus control)**
Normal	10	0	0	0	－
Control	10	5.49	1.81	3.59±1.1	－
Saline	10	5.20	2.61	4.05±0.75	0.141
Vehicle-RNAi	10	5.11	2.01	3.64±1.02	0.867
HIF-1αRNAi-A	10	2.83	0	1.13±0.96	0.000

Neovascularization was scored according to the distance from the limbus to the farthest end of the new vesicles: 0 (no visible vessels in cornea), +1.5 (1/4 distance to center), +2 (1/3 distance to center), +3 (1/2 distance to center), +4 (2/3 distance to center), +4.5 (3/4 distance to center) and +6 (vessels reach center).

### *HIF-1α* shRNA decreased the corneal expression of angiogenic factors and nuclear factor-κB pathway activity in CL wear corneas

**Figure 6:**
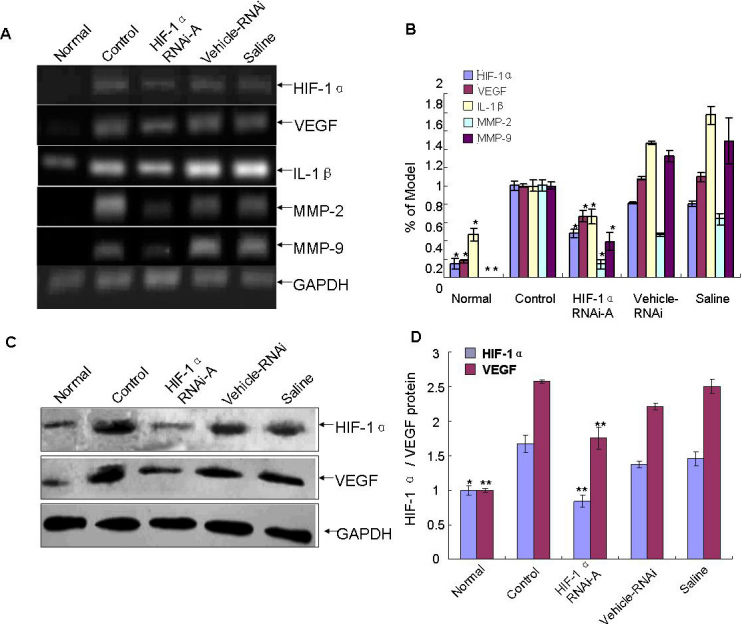
Effects of *HIF-1α* shRNA on *HIF-1α*, *VEGF*, *MMP-2/9*, and *IL-1β* expression in corneas with CL. *HIF-1α* and *VEGF* were detected by RT–PCR (**A**) and western blotting (**C**) while *MMP-2/9* and *IL-1β* were detected by RT–PCR (**A**). GAPDH was used as the internal control in all cases. As shown by the analysis results (**B, D**) compared with the control group, *HIF-1α* RNAi-A significantly down-regulated the expression levels of *VEGF*, *MMP-2/9*, and *IL-1β*. Three independent experiments were conducted, and data were shown as mean±SD *p<0.05, **p<0.01 as compared with control group.

**Figure 7:**
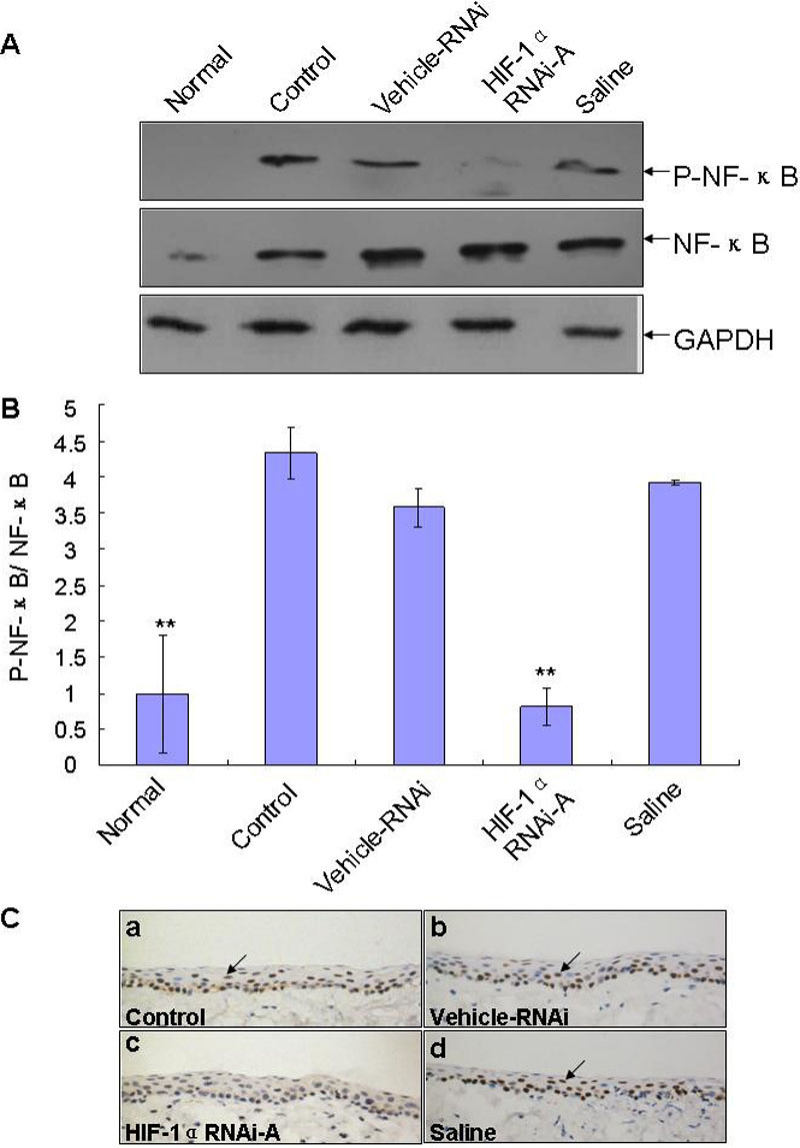
Measurement of nuclear factor-kappa B (NF-κB) pathway proteins by western blotting in corneas. **A**: Total lysates from murine corneal tissue lysates were analyzed by western blotting for their level of NF-κB activation by detecting phosphorylated NF-κB (P-NF-κB), and total NF-κB protein. As shown by the analysis results (**B**), HIF-1α RNAi-A significantly down-regulated NF-κB phosphorylation expression. Three independent experiments were conducted, and data were shown as mean±SD **p<0.01 as compared with the Vehicle-RNAi group. **C**: Immunohistochemistry staining of P-NF-κB in corneas with CL. The arrows indicated the positive staining of P-NF-κB in the corneal epithelium of each group. P-NF-κB was located to the nucleus. Magnification: 400×.

The effect of shRNA treatment on *HIF-1α* expression was assessed by RT–PCR and western blotting analysis. As seen in Figure 6A,C, *HIF-1α* RNAi-A indicated a significant inhibition of *HIF-1α* mRNA and protein expression in *HIF-1α* RNAi-A treated eyes. Expression of *VEGF*, *MMP-2/9*, and *IL-1β* in corneas was measured by using western blotting or RT–PCR. We found that *HIF-1α* RNAi-A effectively inhibited *VEGF*, *MMP-2/9*, and *IL-1β* expression in the CL wear model (Figure 6). Lastly we examined the activity of NF-κB pathway and found that *HIF-1α* RNAi-A significantly reduced NF-κB phosphorylation without changing the total amount of NF-κB (Figure 7).

## Discussion

Contact lenses interact mechanically with the cornea and modify the physiologic processes of corneal tissue. These changes may lead to reduced corneal function [18]. It is necessary to differentiate corneal changes that are physiologically acceptable from those that are pathologic. Clinicians, biochemists, engineers, and vision scientists have performed countless research studies to understand the etiology of these changes to enhance the safety of CL wear. The major consequence of CL wear is chronic hypoxia, with corresponding hypercapnia [19].

Fitting a CL on the eye leads to a significant reduction in the oxygen supply to the cornea, in the range of 8% to 15%, depending on the gas permeability of the lens material used. After prolonged corneal hypoxia, there is depletion of the glycogen reserves of the cornea, diminished ATP (ATP), and ultimately a slowing of the water transport system in the endothelium. The combined effects of the accumulation of lactic acid in the stroma and a decrease in the pumping action of the endothelium result in increased corneal edema.

Corneal NV is a normal (albeit undesirable) vascular response to CL wear, and some vascular response occurs with almost all CL. There are several steps in the neovascularization process: (1) limbal hyperemia, a dilatation of existing limbal capillaries, is reversible and common; (2) superficial neovascularization (pannus) is the progression of limbal hyperemia and the penetration of vessels into the superficial cornea; (3) deep stromal neovascularization results from chronic hypoxia that may progress to an active inflammatory or fibrovascular deep pannus; and (4) there may be an intracorneal hemorrhage [19].

Although corneal NV has been reported during extended wear of disposable and conventional lenses, quantitative and comparative data are lacking. It is especially common with large and thick CL and results in development of new corneal vessels in up to 20% of wearers [19]. No single theory can account for corneal NV; rather, several factors may contribute [20]. Proposed theories take the following aspects into account: metabolic factors (hypoxia, lactic acid, edema); angiogenic suppression; vasostimulation; and neural control. One or all of these stimuli are present during CL wear, particularly overnight wear. Whatever classification system is used, the most important factor is CL-induced tissue hypoxia.

*HIF-1α* is regulated through ubiquitin-mediated degradation under normoxic conditions and stabilization and activation under conditions of hypoxia. It is well known that under hypoxic condition, binding of HIF-1α to the hypoxia response element (HRE) of VEGF promoter results in transcription activity [21-23]. Therefore, we determined the time dependent expression of HIF-1α and correlated it to that of VEGF expression in the mouse model of closed eye CL-induced injury. In this model, hypoxia is believed to be the principle contributor to adverse effects of CL wear [4,24]. Our results showed that mRNA and protein levels of *HIF-1α* in the cornea epithelium of CL wear in response to hypoxia, followed by increasing expression of *VEGF*. mRNA and protein levels of *HIF-1α* and *VEGF* decreased dramatically after transfection with an *HIF-1α* specific shRNA vector.

Based on these results, we presumed that hypoxia may mediate corneal NV through HIF-1α-regulated VEGF in this model. To further elucidate the mechanism for this, we investigated which related factors were involved in regulating corneal NV. Western blotting and RT–PCR assay showed that such antiangiogenic activity might involve a decrease of *IL-1β* and *MMP-2/9* expression. As a principal degrader of extracellular matrix (ECM), MMP-2/9 were proposed to facilitate the growth of new blood vessels by breaking down the ECM and amplifying effect of other angiogenic factors [25], thus representing a logical target for anti-angiogenic therapy. IL-1β is one of the key mediators involved in many inflammatory responses [26-28].

On the other hand, NF-κB plays an important role in IL-1β related inflammatory diseases, including various corneal diseases [29,30]. NF-κB is an important therapeutic target in chronic inflammatory diseases, enabling significant downregulation of macrophage-produced proinflammatory cytokines [31-33]. There are conflicting data in the literature regarding the role of NF-κB in VEGF signaling. One group has been unable to demonstrate an effect of the NF-κB pathway on the expression of VEGF. Others have shown that the upregulation expression of VEGF is regulated by increased NF-κB activity [34,35]. Signaling via the NF-κB pathway has recently been suggested to play a role in the transcriptional regulation of the VEGF gene in various cancer cells [36,37]. In addition, administration of NF-κB antisense oligonucleotides partially inhibited the TNFα and platelet-activating-factor-dependent production of VEGF in human vascular endothelial cells [38].

In summary, our study directly confirmed that inhibition of HIF-1α expression attenuated corneal NV induced by prolonged CL wear. The results strongly implicated corneal HIF-1α as a component of the inflammatory and neovascular cascade initiated by CL wear. Recent interest in inhibiting neovascularization has focused on VEGF [39]. Together with the results presented here, raised the possibility of a new approach to the problem of preventing corneal NV that targets the HIF-1α system. Preliminary findings also supported a functional role for NF-κB in the VEGF signaling pathway, but more studies in depth should be done to expound the exact mechanisms for such gene therapy.
